# Pre-Transplant Plasma Torque Teno Virus Load and Increase Dynamics after Lung Transplantation

**DOI:** 10.1371/journal.pone.0122975

**Published:** 2015-04-20

**Authors:** Irene Görzer, Peter Jaksch, Michael Kundi, Tamara Seitz, Walter Klepetko, Elisabeth Puchhammer-Stöckl

**Affiliations:** 1 Department of Virology, Medical University of Vienna, Vienna, Austria; 2 Division of Thoracic Surgery, Medical University of Vienna, Vienna, Austria; 3 Institute of Environmental Health, Medical University of Vienna, Vienna, Austria; Kliniken der Stadt Köln gGmbH, GERMANY

## Abstract

**Background:**

The human Torque Teno virus (TTV) causes persistent viremia in most immunocompetent individuals. Elevated TTV levels are observed after solid organ transplantation and are related to the extent of immunosuppression especially during the phase of maintenance immunosuppression. However, the extent to which the TTV increase in the early phase post-transplantation is associated with the patient’s immunosuppressive state is unclear.

**Objectives:**

In this study, we assessed the TTV increase dynamics in detail during the first three months after lung transplantation under a defined immunosuppressive regimen and in relation to the pre-transplant TTV level.

**Study Design:**

Forty-six lung transplant recipients (LTRs) were included in this prospective longitudinal study. All received alemtuzumab induction combined with tacrolimus and corticosteroids immunosuppressive therapy. Plasma TTV DNA was monitored before transplantation and regularly within the first three months post-transplantation (n = 320 samples; mean sampling interval: 12.2 days).

**Results:**

In 43/46 LTRs (93%), TTV DNA was detectable before transplantation (median 4.4 log_10_ copies/mL; range: 2.0–6.4). All 46 LTRs showed a TTV increase post-transplantation, which followed a sigmoidal-shaped curve before the median peak level of 9.4 log_10_ copies/mL (range: 7.6–10.7) was reached at a median of day 67 (range: 41–92). The individual TTV DNA doubling times (range: 1.4–20.1 days) significantly correlated with the pre-transplant TTV levels calculated over 30 or 60 days post-transplantation (r = 0.61, 0.54, respectively; both *P* < 0.001), but did not correlate with the mean tacrolimus blood levels. Pre-transplant TTV levels were not associated with time and level of the patients’ post-transplant TTV peak load.

**Conclusion:**

The TTV level may be used to mirror the state of immunosuppression only after the patients’ initial peak TTV level is reached.

## Introduction

The human Torque Teno virus (TTV), a small non-enveloped single-stranded DNA virus, is highly prevalent in humans, but to date, no clear association of TTV with a specific human illness has been found [1,2]. TTV DNA in plasma is detectable in up to 90% of healthy, non-immunocompromised people [2–4]. Hematopoietic stem cells play a pivotal role in maintaining the plasma TTV viremia [5], and activated peripheral blood mononuclear cells also appear to be a main source of TTV DNA in plasma [6,7].

The plasma TTV DNA level in healthy people and in non-immunocompromised patients ranges between 2 and 6 log_10_ copies/mL and reflects the balance between virus replication and antiviral immune response, with an estimated daily clearance rate of more than 90% of virions [3,8–10]. In non-immunocompromised people, TTV remains at a steady-state equilibrium over years, and the TTV level seems to be influenced by age, gender and HCMV seropositivity as recently shown [4]. TTV has been shown to elicit humoral and innate immune responses [11–13] and to influence cytokine production and secretion [11,14,15]. The plasma TTV DNA level is significantly elevated under drug-induced immunosuppression and in patients with low CD4^+^ T cell counts, and thus cell-mediated immunity is considered to play an important role in controlling the TTV plasma level [3,16–19].

Recent studies have shown that the plasma TTV level in solid organ transplant patients is strongly increased upon drug-induced immunosuppression followed by a continuously high steady-state level that appears to correlate with the intensity of immunosuppression [3,18–21]. Furthermore, lower TTV levels in the follow-up are significantly associated with signs of transplant rejection and higher TTV levels are significantly associated with the occurrence of infection episodes [18–20]. Thus, whether regular TTV load measurements after transplantation might become a useful tool for monitoring the strength of immunosuppression remains an important topic of investigation.

While it has repeatedly been shown that the initiation of immunosuppressive therapy causes a substantial increase of the plasma TTV level, the TTV increase dynamics during the early phase after transplantation has not been well investigated. A recent study showed that the administration of a T-cell depleting induction therapy could lead to a temporary drop in the TTV load during the first 15 days post-transplantation [22]. Thus far, to what extent the TTV DNA kinetics over the first three months post-transplantation is associated with the state of immunosuppression is still unclear. Furthermore, whether the TTV DNA load before transplantation has an impact on the TTV DNA load dynamics following initiation of immunosuppressive therapy after lung transplantation is also unknown.

In the present study, we examined the TTV DNA dynamics within the first weeks post-transplantation in lung transplant patients receiving a defined protocol of induction and maintenance immunosuppressive regimen to explore the association between pre-transplant plasma TTV load and the post-transplant TTV DNA increase kinetics. We demonstrate that the TTV DNA increase rate appears to be inversely correlated with the pre-transplant TTV plasma level and that similarly high TTV levels are achieved in patients independent of their individual pre-transplant TTV load.

## Materials and Methods

### Patients

In this prospective longitudinal study, a total of 65 adult lung transplant recipients who received a lung transplant at the Medical University of Vienna between April 2013 and March 2014 and who had completed a follow-up of at least three months were enrolled.

All patients underwent a defined immunosuppressive treatment scheme, consisting of induction therapy with alemtuzumab (Campath-1H; Berlex, Montville, NJ) in combination with tacrolimus (Prograf, Astellas Pharma, Austria) and corticosteroids as maintenance immunosuppression. The alemtuzumab dose was 30 mg and was given as a single dose. Tacrolimus target trough levels were 10–12 ng/mL within the first three post-operative months. Prednisolone was started on day one post-operatively at 1 mg/kg/day, and then tapered to 0.2 mg/kg/day within the first three months.

All patients received antibiotic prophylaxis for two weeks post-operatively, anti-fungal prophylaxis with aerosolized amphotericin B during the three-month follow-up, and antiviral prophylaxis with intravenous ganciclovir for two weeks followed by an oral therapy with valganciclovir until the end of the three-month follow-up period, and intraveneous HCMV hyperimmunglobulin (1 mL/kg) on days 0, 7, 14, and 21 post-transplantation (Cytotect, Biotest; Biotest Austria GmbH, Wien, Austria). Furthermore, each patient received *Pneumocystis carinii* lifelong prophylaxis with co-trimoxacole.

Patients were regularly monitored for plasma TTV DNA load during the follow-up, on average twice monthly. Of the 65 patients, 19 patients were excluded from further analyses due to the lack of a TTV DNA load measurement before transplantation (n = 13), or because less than five regular TTV DNA measurements were available post-transplantation (n = 6). From the remaining 46 LTRs, clinical and laboratory data were obtained from medical records and included age, gender, underlying disease, and tacrolimus blood drug levels (S1 and S3 Tables). The median patient age was 54 years (range: 20–66 years) and included 26 females and 20 males. The underlying diseases were pulmonary fibrosis (n = 9), chronic obstructive pulmonary disease (n = 22), cystic fibrosis (n = 5), sarcoidosis (n = 1), alpha1-antitrypsin deficiency (n = 3), bronchiectasis (n = 1), and pulmonary hypertension (n = 5). The patients’ mean tacrolimus target trough levels ranged between 7.8–14.0 ng/mL during the first 30 days, and between 8.4–14.0 ng/mL during 60 days post-transplantation.

#### Ethics statement

The study was conducted at the Department of Virology of the Medical University of Vienna, and the research protocol was approved by the ethics committee of the Medical University of Vienna (EK: 251/2007 and EK: 1710/2014). All patients gave their written informed consent.

### Measurement of plasma TTV DNA load

TTV DNA load measurement from plasma was performed from three days before transplantation to the day of transplantation, and at regular intervals in the course of the patients’ post-transplantation follow-up. Freshly withdrawn plasma samples were processed after receipt within the laboratory. Briefly, TTV DNA was extracted from 200 μL of plasma using the NucliSENS easyMAG platform (bioMerieux, France) as recommended by the manufacturer and eluted in 50 μL of elution buffer. TTV DNA was quantitated by TaqMan real-time PCR as described previously [23]. Results were recorded in log_10_ copies/mL. TTV DNA quantitation was in the linear range from 2 to 10 log_10_ copies/mL as determined by the use of 10-fold dilutions of a plasmid standard. Limit of detection was 2 log_10_ copies/mL of plasma.

### Data analysis

TTV DNA loads were plotted versus post-transplantation time and the best-fitted line was derived based on a variable slope equation without constraints to describe the overall TTV DNA kinetics post-transplantation. TTV DNA doubling time (T2) was determined from each patient separately using the slope of a line fitted by log-linear regression of the virus loads versus on days after transplantation up to the time point when the maximum was reached. Doubling times were calculated using the standard exponential function as previously described [24], with T2 = ln2/Slope.

The relationships between the pre-transplant virus load and the virus load change up to day 15, the TTV DNA doubling time, and the peak virus load, respectively, were determined using Spearman’s correlation coefficient. TTV DNA doubling times between patient groups were compared by Mann-Whitney U test. All *P* values <0.05 were considered significant. GraphPad Prism version 5.0 was used for data analysis.

## Results

All 46 adult LTRs included in this study had a TTV DNA load measurement before transplantation and at least five regular TTV DNA measurements post-transplantation. In 43/46 LTRs (93%), TTV DNA was already detectable at or before transplantation with a median TTV DNA level of 4.4 log_10_ copies/ml (range: 2.0–6.4). During the three months after transplantation of all patients, 320 plasma samples were tested for TTV DNA load (S2 Table), with a mean of 7 post-transplant samples per patient (range: 5–10) and a mean sampling interval in the individual patients of 12.2 days (range: 1–40 days).

**Fig 1:**
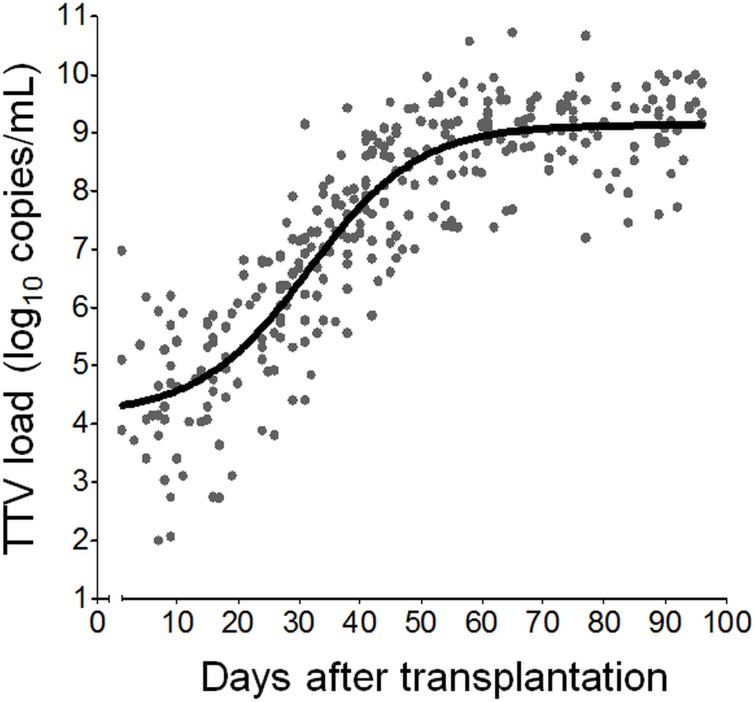
Overall plasma TTV DNA kinetics post-transplantation. Forty-six lung transplant recipients were regularly monitored for plasma TTV DNA load during the first three months post-transplantation with a mean sampling interval of 12.2 days. The individual TTV DNA measurements (n = 320) are represented as filled circles and the computed non-linear regression line of best-fit is shown.

The TTV DNA levels measured in all 46 patients are shown in Fig 1, and the overall DNA kinetics was calculated. The non-linear regression analysis (best-fitted curve) showed that the TTV DNA increase upon initiation of drug-induced immunosuppression followed a sigmoidal-shaped or tri-phasic curve, starting with a slow increase up to around day 15 (delay phase) which then led to a severe, almost linear, increase up to day 45 (exponential phase). From day 45 onwards, the increase slowed down (deceleration phase) and peaked at around day 60 post-transplantation.

### Individual TTV increase kinetics during the first three months post-transplantation

**Fig 2:**
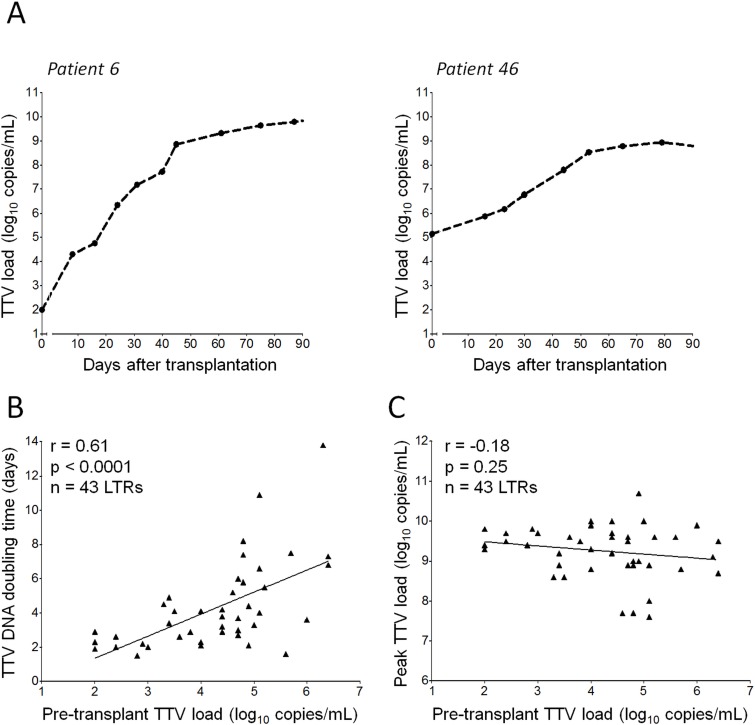
Individual TTV DNA increase kinetics post-transplantation in relation to the pre-transplant TTV level. (A) Individual plasma TTV DNA load increase kinetics after lung transplantation and initiation of immunosuppressive therapy are shown for two lung transplant patients. The individual TTV DNA measurements are represented as filled circles. (B, C) The relationship between the patients’ pre-transplant TTV DNA load and the TTV DNA doubling time over the first 30 days post-transplantation (B), or the patients’ peak TTV DNA level (C) is shown. Correlation analyses were performed by Spearman’s rank test. Tx, transplantation; LTRs, lung transplant recipients.

We further investigated the individual TTV DNA kinetics. In all 46 patients, the TTV DNA increased during the first three months post-transplantation independent of whether the patients were TTV DNA positive or negative before transplantation. The individual peak TTV DNA levels ranged between 7.6 and 10.7 log_10_ copies/mL (median: 9.4) and were reached between 41 and 92 days post-transplantation (median: 67 days). Representative TTV increase curves from two patients are presented in Fig 2A.

Next, we determined the TTV DNA doubling time from the slope of the regression line fitted to the regularly measured TTV DNA loads within the early period after transplantation. The median TTV DNA doubling time was 3.4 days (range: 1.4–13.8 days; n = 46 LTRs) within the first 30 days, and 6.5 days (range: 2.0–20.1 days; n = 41 LTRs) when estimated over the first 60 days post-transplantation (S1 Table).

We then assessed whether the overall TTV DNA doubling time was associated with the pre-transplant TTV load. Statistical analysis by Spearman’s rank test showed that the TTV DNA doubling times calculated for 30 or 60 days post-transplantation significantly correlated with the pre-transplant TTV DNA levels (r = 0.61, 0.54, respectively; both *P* < 0.001). Fig 2B shows the correlation over the first 30 days post-transplantation. Further, we analyzed whether there was a potential relationship between the pre-transplant TTV load and the peak level reached by the patients within the three-month follow-up. No significant association was found between the pre-transplant level and the maximum TTV DNA peak level (Spearman’s r = -0.18; Fig 2C).

Next, we investigated whether the TTV DNA doubling time was associated with the patient tacrolimus trough level. The patients’ mean tacrolimus trough levels over the respective post-transplant periods of 30 and 60 days were determined and compared to the TTV doubling times (S1 Table). No significant correlation between TTV DNA doubling times and the respective mean tacrolimus blood drug level was observed, as revealed by Spearman’s rank test (r = 0.005 and -0.049 for 30 and 60 days, respectively). In addition, associations between the TTV doubling time and age, sex, or underlying disease of the patients were analyzed, but no significant differences were found (Mann-Whitney U test).

### Change in TTV DNA load up to day 15 post-transplantation

**Fig 3:**
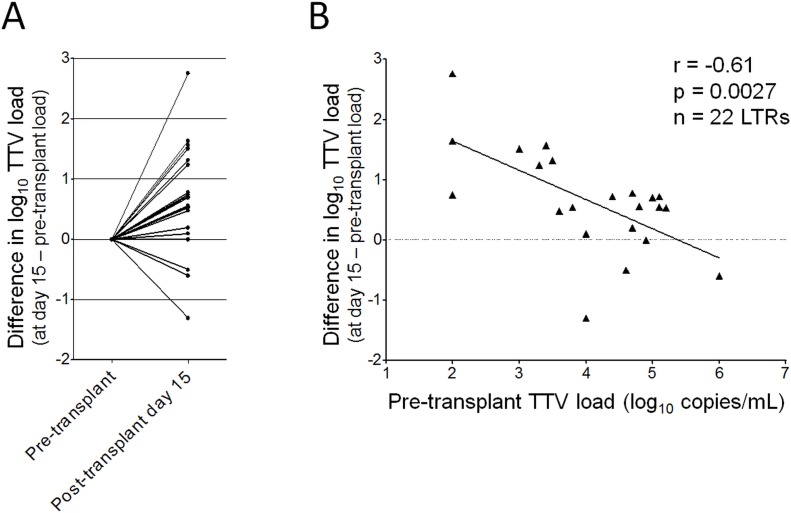
Development of TTV DNA load up to day 15 post-transplantation. (A) Change in log_10_ TTV DNA load from pre-transplant TTV load to day 15 (± 3 days) post-transplantation in plasma of 22 lung transplant recipients. (B) Relationship between pre-transplant TTV load and the change in TTV DNA load up to day 15 analyzed by Spearman’s rank test. LTRs, lung transplant recipients.

Based on the finding that the overall TTV increase seems to start with a delay of around 15 days following initiation of immunosuppression, we aimed to assess the TTV load development within the first 15 days post-transplantation in more detail. Therefore, all LTRs were selected who had a positive pre-transplant TTV DNA load and for whom a TTV DNA measurement at day 15 (± 3 days) post-transplantation was available (n = 22; S4 Table). From these data, the difference between the pre-transplant level and the day 15 TTV level was estimated. As shown in Fig 3A, there was a substantial variation in the TTV load development, ranging from a decline of more than 1 log_10_ copy/mL up to an increase of more than 2 log_10_ copies/mL.

Finally, we investigated whether the change in the TTV DNA load from pre-transplant to day 15 was associated with the pre-transplant TTV load. Statistical analysis by Spearman’s rank test revealed a highly significant inverse correlation between the TTV DNA change and the pre-transplant level (r = -0.61; *P* = 0.0027; Fig 3B).

## Discussion

In the last years different studies have shown that the human TTV load reflects the balance between TTV replication and antiviral immune response [25] and may therefore be a useful tool to indirectly observe the extent of immunosuppression after transplantation [16–21]. Recent data have shown that the plasma TTV load was constantly elevated in lung and heart transplant patients after 2 to 3 months post-transplantation and the individual TTV level was significantly related to occurrence of episodes of infection or rejection [18,20]. In contrast, the TTV load kinetics within the first weeks of immunosuppressive therapy after transplantation has been unclear.

In this prospective study, analyzing this early phase after transplantation we have shown that in lung transplant patients who were treated with alemtuzumab induction therapy and a combination of tacrolimus and steroids, initiation of immunosuppression was followed by a tri-phasic and sigmoidal-shaped course of TTV DNA kinetics. The first remarkable finding was that the TTV increase started in overall in the patients only with a delay of about 15 days after onset of immunosuppression, followed by a strong and almost linear increase between day 15 and day 45 post-transplantation. A second important finding was that the patients’ pre-transplant plasma TTV load inversely correlated with the individual TTV DNA increase rates after transplantation. This provides evidence that higher initial pre-transplant patient plasma TTV levels, although probably representative for a person’s higher infection rate, do not result in a stronger TTV increase upon immunosuppression. In fact, in all patients, independent from their pre-transplant TTV load, the peak of the TTV increase was reached at around day 60 and the overall TTV peak level was also quantitatively not associated to the pre-transplant level.

Several different, but not mutually exclusive, hypotheses may account for the unexpected findings. First, since TTV is a T-lymphotropic virus [22], it is likely that the rapid and effective depletion of peripheral T-lymphocytes by the induction drug alemtuzumab [26] leads to a substantial reduction in the number of TTV infected cells in the peripheral blood. This may explain the delay in TTV DNA increase in the first days post-transplantation despite the rapid impairment of T-cell specific immunity under the given immunosuppressive regimen. A similar depletion effect on the plasma TTV level has been recently demonstrated for two other anti-T lymphocyte drugs, ATG and basiliximab [22]. Since the T cell recovery after alemtuzumab induction takes several months [27], it is, however, not clear why the depletion effect seems to affect the plasma TTV level only very early post-transplantation. It is plausible that the ongoing immunosuppression may facilitate a widening of the host cell range and/or allows a higher replicative activity of the infected cells, and this might finally replace the loss in TTV-infected T cells. However, this does not fully explain why patients with a lower pre-transplant TTV burden may better overcome the loss in TTV-infected cells as these patients showed higher TTV increase rates.

Second, it is possible that a higher pre-transplant TTV level not only shows a higher infection rate but may also reflect an increased inflammatory status similarly, as recently discussed [4]. Dysregulated processes of inflammation are thought to compromise the regenerative capacity of hematopoietic stem cells (HSCs) [28], and HSCs, in turn, are supposed to maintain the plasma TTV level [5]. Thus, the higher the level of basal inflammation in a patient, the lower the regeneration capacity of the T-cell pool, and this may explain why patients with higher pre-transplant TTV levels show lower TTV increase rates in the follow-up.

Third, a higher exposure of TTV to the host as represented by a higher plasma TTV level might lead to a higher expansion of TTV-specific immune cells, as described for other chronic viral infections, particularly for human cytomegalovirus [29]. Effector memory T cells could play an important role in this regard, since these cells are not only resistant to alemtuzumab depletion, but also show proliferation after alemtuzumab-mediated depletion [30]. These effector memory T cells are, however, sensitive to tacrolimus, and the ongoing tacrolimus-based maintenance therapy might result in the later impairment of this alemtuzumab-resistant T-cell subset. This may explain the sigmoidal-shaped TTV increase curve, which appears to reflect a more gradual rather than a rapid impairment of the TTV-specific immune response. Also, our data clearly showed that an influence of the pre-transplant TTV level is no longer visible when the peak TTV level is reached after about 60 days post-transplantation, suggesting a maximally reached impairment of the TTV-specific immune response in all patients. The underlying processes that might account for these hypotheses, however, are poorly understood and need to be elucidated in future studies.

In conclusion, our data further contribute to the knowledge about TTV and TTV kinetics, which is especially important when the extent of TTV replication is considered as a possible tool to mirror the extent of post-transplantation immunosuppression. Our results suggest that the TTV load may be useful for this purpose, starting from when the TTV peak level is reached, which is in average at about 60 days post-transplantation, as this level is not individually associated with pre-transplant virus load. The kinetics of the TTV load early after start of immunosuppression post-transplantation is a complex process which is also associated with pre-transplant TTV load. Further studies will be needed to analyze the functional mechanisms causing the specific TTV kinetics early after immunosuppression.

## Supporting Information

S1 Table Patient characteristics, virological and laboratory parameters. (XLSX)

S2 Table Plasma TTV DNA measurements during the first three months post-transplantation in 46 lung transplant recipients. (XLSX)

S3 Table Tacrolimus blood drug level measurements during the first three months post-transplantation in 41 lung transplant recipients. (XLSX)

S4 Table Plasma TTV DNA load before transplantation (Pre-Tx) and at day 15 (+/- 3 days) post-transplantation (Post-Tx) in 22 lung transplant patients. (XLSX)
